# Production of camptothecin in cultures of *Chonemorpha grandiflora*

**DOI:** 10.4103/0974-8490.72327

**Published:** 2010

**Authors:** A. V. Kulkarni, A. A. Patwardhan, U. Lele, N. P. Malpathak

**Affiliations:** *Department of Botany, University of Pune, Pune -411007, Maharashtra, India*; 1 *Department of Microbiology, M. E. S. Garware College, Pune -411007, Maharashtra, India*

**Keywords:** Apocynaceae, callus, camptothecin, *Chonemorpha grandiflora*

## Abstract

**Background:**

*Chonemorpha grandiflora* (Syn. Chonemorpha fragrans (Apocynaceae) is an endangered medicinal plant. It is used in different preparations, such as sudarsanasavam and kumaryasavam used in Kerala Ayurvedic system. *C. grandiflora* is used for the treatment of fever and stomach disorders. Phytochemical investigations have revealed the presence of steroidal alkaloids, such as chonemorphine and funtumafrine in *C. grandiflora*. Camptothecin, a well-known anticancer alkaloid has been detected in ethanolic extracts of stem with bark and callus cultures derived from *C. grandiflora*.

**Methods:**

Callus cultures of *C. grandiflora* were raised on Murashige and Skoog’s medium supplemented with 2, 4-D. Stem with bark and callus were used for phytochemical analysis mainly the alkaloids. Detection and identification of camptothecin was carried out using thin-layer chromatography (TLC), high-performance thin-layer chromatography, (HPTLC) and high-performance liquid chromatography (HPLC).

**Results:**

An important anticancer alkaloid, camptothecin was detected in ethanolic extracts of stem with bark and callus cultures of *C. grandiflora*. camptothecin content was 0.013 mg/g in stem with bark and 0.003 mg/g in callus.

**Conclusion:**

This is the first report on *in vivo* and *in vitro* production of camptothecin in *C. grandiflora*. Camptothecin is known to occur only in six plant sources so, alternative sources for camptothecin are needed. Thus of *C. grandiflora* could be a new promising alternative source of camptothecin.

## INTRODUCTION

*Chonemorpha grandiflora*(Roth) M. R. and S. M. Almeida (Syn. *Chonemorpha fragrans*) is a shrubby, latex-bearing climber belonging to the family Apocynaceae. It is a medicinal plant,[1,2] which has been assigned endangered status in Karnataka state and vulnerable in Kerala state.[3] It is used in different preparations, such as sudarsanasavam and kumaryasavam used in Kerala Ayurvedic system.[4] It is used for the treatment of fever and stomach disorders. Entire plant, roots, and root-bark are used for the treatment. The trade is mainly confined to Kerala state under the name Perumkurumba and the dried roots are sold commercially.[5] *C. grandiflora* is shown to possess antiparasitic and muscle relaxant properties.[6,7] Phytochemical investigations have revealed the presence of steroidal alkaloids, such as chonemorphine and funtumafrine.[8,9] There are so far no reports on phytochemical investigations of *in vitro* material of *C. grandiflora*. Thus, cultures of *C. grandiflora* were studied for the production of alkaloids and compared with *in vivo* material.

## MATERIAL AND METHODS

### Establishment of callus cultures

The plant material was obtained from Kerala (Thrissur district). The voucher specimens were identified and submitted at Botanical Survey of India, Western Circle, Pune, India. The plants were established by cutting and maintained in the Botanical garden, Department of Botany, University of Pune. Internodes from these plants were used for initiation and establishment of callus cultures in *C. grandiflora*. The intermodal segments were sterilized by using 0.1% HgCl_2_ and 70% alcohol and grown on Murashige and Skoog medium[10] supplemented with 4.52 μM 2,4-dichlorophenoxyacetic acid to raise callus cultures. Callus cultures were subcultured every fourth week.

### Phytochemical analysis

The callus and the stem with bark was shade dried and used for phytochemical analysis. The plant material was powdered and used for the preparation of ethanolic extracts. Cold extraction was carried out using 50 g of the powder of the plant material and 200 mL ethanol for 48 h. The extracts were centrifuged at 9000 *g* for 5 min. The clear supernatant was passed through the membrane filter (cellulose nitrate, 0.20 μm, Pall Gellman, Bombay, India). The extracts were evaporated to dryness to get the residue. To the residue, 1 mL of methanol was added and these samples were used for thin-layer chromatography (TLC), high-performance thin-layer chromatography (HPTLC), and high-performance liquid chromatography (HPLC) analysis.

- TLC was performed on silica gel 60 F_254_ precoated (20 × 20 cm; Merck, Darmstadt, Germany) plates, using protocol described by Fulzele *et al*. (2001).[11] A pure sample of camptothecin was procured from Sigma Aldrich, Bangalore. A standard sample of camptothecin was prepared by dissolving 40 μg camptothecin in dimethyl sulfoxide (DMSO):methanol (1:50) and run along with the extracts. Rf of standard camptothecin was recorded.
- For HPTLC analysis, 500 μg of the extracts were loaded on HPTLC plates. The plates were run in duplicates in solvent systems (i) ethyl acetate:toluene (7:3) and (ii) chloroform:ethyl acetate (1:1). The chromatographs were scanned by Camag densitometric scanner and the peaks, peak areas, and the Rf of the spots were recorded. A pure sample of camptothecin was procured from Sigma Aldrich, Bangalore. A standard sample of camptothecin was prepared by dissolving 40 μg of camptothecin in 1 mL of DMSO:methanol (1:50) and run along with the extracts. Fluorescence was recorded at 366 nm. Rf of the standard camptothecin was recorded.
- Isocratic analytical HPLC was carried out using RP-C18 column (Perkin Elmer, series 200, Switzerland, SPHERI-5, 5 mm, 250 × 4.6 mm). The mobile phase for alkaloid elution was acetonitrile:water (40:60), at a flow rate 1.6 mL/min with a sample size of 20 μL; and UV detection at 254 nm. A standard curve was obtained using authentic sample of camptothecin (Sigma Aldrich). The standard was prepared using DMSO:methanol (1:50 v/v). HPLC analysis of standard as well as extract yielded chromatogram with retention time of 3.85 min. Co-chromatography of the extracts was performed with authentic samples for confirmation. Validation of quantitative method was performed for samples in 5 replications. The results from the samples at two concentrations did not alter the retention time. The retention time proved that accuracy and reproducibility was excellent.

**Figure 1:**
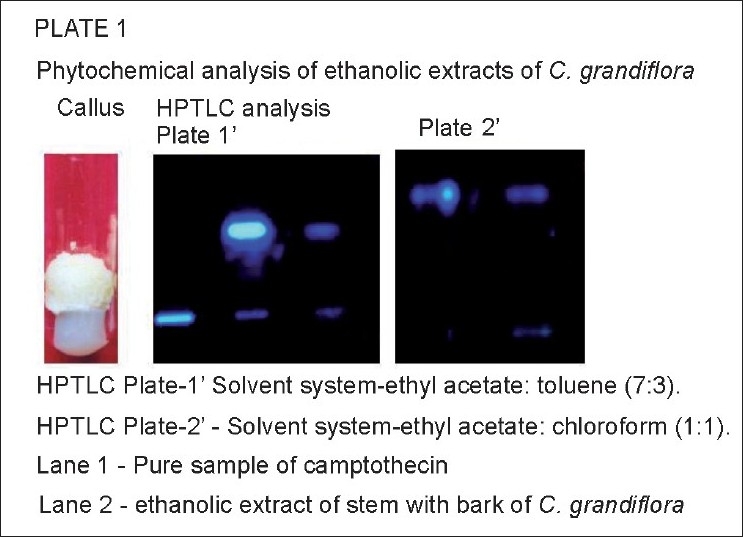
HPTLC analysis of ethanolic extracts of *Chonemorpha grandiflora*.

## RESULTS

**Figure 2:**
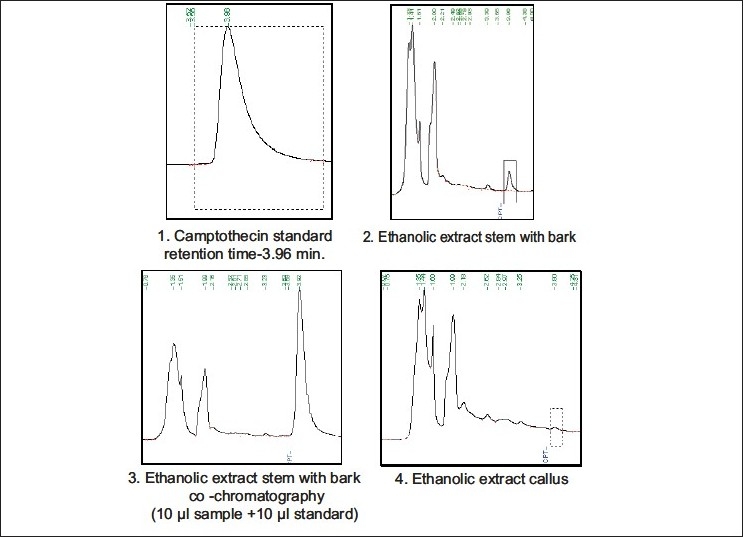
HPLC analysis of ethanolic extracts of *Chonemorpha grandiflora*.

- TLC analysis—Camptothecin showed a dark blue spot at 254 nm and Rf value was 0.46 in solvent system chloroform:ethyl acetate (1:1). In ethanolic extracts of stem with bark of *C. grandiflora*, a very faint spot with the same Rf and blue fluorescence at 254 nm were observed.
- HPTLC analysis revealed the presence of a compound having same Rf as that of standard camptothecin in the ethanolic extract of stem with bark of *C. grandiflora* [Figure 1; Plate 1.].
- (c) HPLC analysis also showed the presence of a peak having same retention time as that of pure camptothecin in the ethanolic extracts of stem with bark and callus of *C. grandiflora* [Figure 2; Plate 2]. The amount of camptothecin in the samples was calculated considering the following values: (1) peak area shown by standard camptothecin sample, (2) peak area of peak in plant extracts showing the same retention time as that of standard camptothecin, (3) total volume of the extract prepared, and (4) dry weight of the plant material used to prepare the extract. Percentage of camptothecin was calculated for the samples on dry weight basis (mg/g). The stem with bark yielded 0.013 mg/g camptothecin, whereas internode callus yielded 0.003 mg/g camptothecin [Figure 2 Plate 2].

## DISCUSSION

Camptothecins are one of the most important anticancer alkaloids of the 21^st^ century because of their clinical applications against cancer[12,13] and HIV.[14] They have been found to be active against parasitic trypanosomes, *Leishmania*,[15] and *falciparum* malaria.[16] Camptothecin is known to occur in different unrelated genera, including *Camptotheca acuminata*,[17] *Nothapodytes nimmoniana*,[18,19] *Tabernaemontana heyneana*,[20] and *Ophiorrhiza rugosa* var. *prostrata*.[21] Camptothecin was detected and identified in ethanolic extracts of stem with bark and callus derived from *C. grandiflora* using TLC, HPTLC, and HPLC. Thus, on the basis of the present investigations, we propose *C. grandiflora* as a new source of camptothecin.

The yield of camptothecin calculated for *C. acuminata* was 400-5000 mg/g,[17] for *N. nimmoniana* 0.23%-0.33%,[19] and for *T. heyneana* stem bark 0.00013%.[20] Ever increasing worldwide demand for camptothecin from pharmaceutical industries and subsequent pressure on the wild populations of *N. nimmoniana* and *C. acuminata* has endangered the plants. Thus, there is an urgent need to find alternative plant sources of camptothecin. Although the amount of camptothecin reported by us in stem with bark in *C. grandiflora* is low as compared with that reported in *C. acuminata* and *N. nimmoniana*, it is more as compared with that reported in *T. heyneana*. Thus, *C. grandiflora* could be a new promising alternative source of camptothecin.

*In vitro* cultures of *C. acuminata*, *N. nimmoniana*, and *Ophiorrhiza pumila* have been established for camptothecin production. Undifferentiated callus cultures[22] and suspension cultures, usually produce significantly low amount of camptothecin, for example, *C. acuminata*[23] and *N. nimmoniana* (0.0003%-0.01%).[24] In callus cultures of N. foetida, camptothecin levels reported were 100- to 1000-fold lower than in the intact plant.[25,26] Root and hairy root cultures of O. pumila have been successfully employed for camptothecin production.[27] Presence of camptothecin has been detected from callus cultures of *C. grandiflora*. Although the amount reported is low, it could be enhanced by using biotic and abiotic elicitors. Thus, our results indicate *C. grandiflora* callus, a new and promising source of camptothecin useful in drug development.
